# Regional variations in Mediterranean diet adherence: a sociodemographic and lifestyle analysis across Mediterranean and non-Mediterranean regions within the MEDIET4ALL project

**DOI:** 10.3389/fpubh.2025.1596681

**Published:** 2025-06-10

**Authors:** Mohamed Ali Boujelbane, Achraf Ammar, Atef Salem, Mohamed Kerkeni, Khaled Trabelsi, Bassem Bouaziz, Liwa Masmoudi, Juliane Heydenreich, Christiana Schallhorn, Gabriel Müller, Ayse Merve Uyar, Hadeel Ali Ghazzawi, Adam Tawfiq Amawi, Bekir Erhan Orhan, Giuseppe Grosso, Osama Abdelkarim, Tarak Driss, Kais El Abed, Piotr Zmijewski, Frédéric Debeaufort, Nasreddine Benbettaieb, Clément Poulain, Laura Reyes, Amparo Gamero, Marta Cuenca-Ortolá, Antonio Cilla, Nicola Francesca, Concetta Maria Messina, Enrico Viola, Björn Lorenzen, Stefania Filice, Aadil Bajoub, El-Mehdi Ajal, El Amine Ajal, Majdouline Obtel, Sadjia Lahiani, Taha Khaldi, Nafaa Souissi, Omar Boukhris, Haitham Jahrami, Waqar Husain, Evelyn Frias-Toral, Walid Mahdi, Hamdi Chtourou, Wolfgang I. Schöllhorn

**Affiliations:** ^1^Department of Training and Movement Science, Institute of Sport Science, Johannes Gutenberg-University Mainz, Mainz, Germany; ^2^High Institute of Sport and Physical Education of Sfax, University of Sfax, Sfax, Tunisia; ^3^Research Laboratory, Molecular Bases of Human Pathology, LR19ES13, Faculty of Medicine of Sfax, University of Sfax, Sfax, Tunisia; ^4^Research Laboratory, Education, Motricity, Sport and Health, EM2S, LR19JS01, High Institute of Sport and Physical Education of Sfax, University of Sfax, Sfax, Tunisia; ^5^Department of Movement Sciences and Sports Training, School of Sport Science, The University of Jordan, Amman, Jordan; ^6^Multimedia InfoRmation Systems and Advanced Computing Laboratory (MIRACL), University of Sfax, Sfax, Tunisia; ^7^Higher Institute of Computer Science and Multimedia of Sfax (ISIMS), University of Sfax, Sfax, Tunisia; ^8^Department of Experimental Sports Nutrition, Faculty of Sports Sciences, Leipzig University, Leipzig, Germany; ^9^Department of Sports Economics, Sociology and History, Institute of Sport Science, Johannes Gutenberg-University Mainz, Mainz, Germany; ^10^Department of Nutrition and Food Technology, School of Agriculture, The University of Jordan, Amman, Jordan; ^11^Faculty of Sports Sciences, Istanbul Aydın University, Istanbul, Türkiye; ^12^Department of Biomedical and Biotechnological Sciences, University of Catania, Catania, Italy; ^13^Faculty of Sport Sciences, Assiut University, Assiut, Egypt; ^14^ESLSCA University Egypt, Giza, Egypt; ^15^Interdisciplinary Laboratory in Neurosciences, Physiology, and Psychology: Physical Activity, Health, and Learning (LINP2), UFR STAPS, Paris Nanterre University, Nanterre, France; ^16^Jozef Pilsudski University of Physical Education in Warsaw, Warsaw, Poland; ^17^Department BioEngineering, Institut Universitaire de Technologie IUT-Dijon-Auexrre-Nevers, University Burgundy Europe, Dijon, France; ^18^Joint Research Unit UMR PAM-Food Processing and Microbiology, Université Bourgogne Europe, INRAE, Institut AgroDijon, Dijon, France; ^19^Vitagora Innovation Cluster, Dijon, France; ^20^Department of Preventive Medicine and Public Health, Food Science, Toxicology and Forensic Medicine, Faculty of Pharmacy and Food Sciences, University of Valencia, Valencia, Spain; ^21^Department of Agricultural Food and Forest Sciences, University of Palermo, Palermo, Italy; ^22^Laboratory of Marine Biochemistry and Ecotoxicology, Department of Earth and Marine Sciences DiSTeM, University of Palermo, Trapani, Italy; ^23^Microtarians SIS, Société d’Impact Societal, Luxembourg, Luxembourg; ^24^Laboratory of Food and Food By-Products Chemistry and Processing Technology, National School of Agriculture in Meknès, Meknès, Morocco; ^25^Laboratory of Social Medicine, Department of Epidemiology and Public Health, Faculty of Medicine and Pharmacy of Rabat, Mohammed V University, Rabat, Morocco; ^26^UPR of Pharmacognosy, Faculty of Medicine and Pharmacy of Rabat, Mohammed V University, Rabat, Morocco; ^27^VALCORE Laboratory, Department of Biology, Faculty of Science, University of M’hamed Bougara Boumerdes, Boumerdes, Algeria; ^28^Biotechnology Research Center C.R.Bt Constantine ALGERIA, Constantine, Algeria; ^29^SIESTA Research Group, School of Allied Health, Human Services and Sport, La Trobe University, Melbourne, VIC, Australia; ^30^Sport, Performance, and Nutrition Research Group, School of Allied Health, Human Services and Sport, La Trobe University, Melbourne, VIC, Australia; ^31^Government Hospitals, Manama, Bahrain; ^32^Department of Psychiatry, College of Medicine and Medical Sciences, Arabian Gulf University, Manama, Bahrain; ^33^Department of Humanities, COMSATS University Islamabad, Islamabad, Pakistan; ^34^Escuela de Medicina, Universidad Espíritu Santo, Samborondón, Ecuador; ^35^Division of Research, Texas State University, San Marcos, TX, United States

**Keywords:** Mediterranean regions differences, MedLife Index, physical activity, mental health, public health, psychological distress, health predictors

## Abstract

**Introduction:**

The Mediterranean diet (MedDiet) is acknowledged for its health advantages; however, compliance with its principles differs by region and is influenced by geographical, cultural, economic, and life-style factors. This research examines regional differences in sociodemographic and lifestyle factors between Mediterranean (MC) and non-Mediterranean (NMC) countries, with a particular focus on adherence to the Mediterranean diet and lifestyle, as well as the associated barriers in each region.

**Methods:**

The MEDIET4ALL international survey was conducted across 10 countries, and data were collected from 4,010 participants. Dietary adherence was assessed via the MedLife Index, and additional lifestyle measures included physical activity (IPAQ-SF), sleep patterns (PSQI), mental health (DASS-21), and social participation (SSPQL). Statistical analyses included chi-square tests, Mann–Whitney U tests, and standardized residual analyses to identify significant regional variations.

**Results:**

The study revealed distinct dietary patterns, with MC participants showing stronger adherence to traditional MedDiet components (legumes, fish) while NMC participants favored modern adaptations (whole grains). Both regions exhibited low physical activities dominance (60%–62%), although MC participants engaged more (21.1% vs. 18.5%) in moderate physical activity. MC maintained higher proportions of “sometimes socially active” individuals, NMC showed greater representation in the “always socially active” category. Sleep quality was poorer in MC (45% below recommended duration vs. 40% in NMC), while NMC reported higher insomnia rates. Mental health symptoms were comparable (33%–35% moderate depression/anxiety in both), reflecting post-pandemic global trends. Barriers differed regionally with MC faced economic/access constraints while NMC struggled with knowledge gaps and time limitations.

**Conclusion:**

Our findings highlight that while Mediterranean regions maintain traditional dietary patterns, globalization and modern lifestyle shifts are narrowing regional health behaviors. Public health strategies should address region-specific barriers, including economic constraints in MC regions and knowledge gaps in NMC regions, while promoting MedDiet adherence. Future research should explore the impact of cultural, socio-economic, and digital factors on dietary behaviors and mental health to develop tailored, effective interventions for improving overall well-being.

## 1 Introduction

The scientific community has increasingly focused on assessing the risk factors associated with dietary practices and their potential associations with public health (1). Current evidence suggests that traditional dietary patterns are undergoing a profound transformation due to globalization and the increased availability of processed, ready-to-eat, high-calorie foods (2). This transition has led to a decline in locally sourced, nutrient-dense foods and an increase in diets characterized by high levels of added sugars, saturated and hydrogenated fats, and a deficiency in fiber and micronutrients. Such dietary changes are linked to the increasing prevalence of noncommunicable diseases (NCDs), including obesity, diabetes, cardiovascular diseases, and certain cancers (3).

While numerous traditional dietary patterns worldwide are associated with health benefits, the MedDiet stands out as one of the most extensively researched (4). Rooted in the eating habits of Greece and Italy during the early 1960s, the MedDiet is characterized by high consumption of olive oil, fruits, vegetables, whole grains, nuts, and legumes; moderate intake of fish and dairy; and low consumption of red meat and processed foods (5). Its nutritional composition is rich in bioactive compounds with antioxidant and anti-inflammatory properties, is associated with its low glycemic index and potential health benefits (6).

Numerous systematic reviews and meta-analyses of population-based and prospective epidemiological studies have demonstrated that adherence to the MedDiet is associated with a reduced risk of obesity (7), hypertension (8), type 2 diabetes (9), metabolic syndrome (10), primary (11) and secondary prevention of cardiovascular diseases (12), certain types of cancer (13), and neurodegenerative disorders, such as cognitive decline and Alzheimer’s disease (14). Besides, higher adherence to this dietary pattern has also been associated with better physical and mental quality of life in the general population and in individuals with a variety of health conditions (15). Finally, the Mediterranean diet is also considered an ideal dietary model supporting the balance among people, environment, and society (16).

Adherence to the MedDiet varies across regions due to geographical, cultural, economic, and lifestyle factors (17). Traditionally, MC including those in Southern Europe (Spain, Italy, France, Turkey), North Africa (Tunisia, Algeria, Morocco), and the Middle East (Jordan), have followed a diet rich in plant-based foods, seafood, and olive oil, which aligns with the MedDiet model. However, globalization and urbanization have led to a shift away from these dietary habits, resulting in the increasing adoption of Westernized diets in these regions (18). This transition coincides with a steady decline in MedDiet adherence, despite its well-documented health benefits (19). Socioeconomic factors play a crucial role in dietary choices, as individuals with higher socioeconomic status are more likely to consume nutrient-dense foods such as fruits, vegetables, and whole grains, whereas those with lower socioeconomic status often rely more on refined grains and processed foods owing to cost and accessibility (20). Additionally, lifestyle behaviors, including smoking and physical inactivity, are linked to lower adherence to the MedDiet, often coinciding with increased fast-food consumption (21). In contrast, non-Mediterranean regions, such as Central and Northern Europe (Germany, Luxembourg), exhibit distinct dietary patterns shaped by local food availability, cultural traditions, and economic conditions. The consumption of diets in these regions tends to be greater for processed foods, red meat, and dairy products, with lower consumption rates of olive oil and fresh produce (22). Nonetheless, the growing global awareness of the health benefits of the MedDiet has led to efforts to promote its adoption in NMC. However, barriers such as food availability, affordability, and ingrained culinary traditions continue to affect the extent to which individuals in these regions can integrate MedDiet principles into their daily eating habits (23, 24). As part of the broader MEDIET4ALL PRIMA project, which is supported by the European Union, this research aligns with the initiative’s goal of promoting the MedDiet and its associated lifestyle as a sustainable and health-focused model (25). This study aims to evaluate regional variations in the MedDiet adherence by analyzing sociodemographic and lifestyle factors across the Mediterranean and non-Mediterranean regions.

## 2 Materials and methods

### 2.1 Survey design and participants recruitment

The MEDIET4ALL survey was conducted as an international online study over a period of 4 months, starting in the summer of 2024, to assess adherence to the Mediterranean Diet across multiple countries and regions. Designed by a multidisciplinary team of specialists in public health, nutrition, psychology, and social sciences, the survey prioritized representativeness in regional comparisons while acknowledging inherent limitations of digital recruitment (e.g., higher participation from younger, urban populations). The study targeted participants from Tunisia, Algeria, Morocco, France, Spain, Germany, Luxembourg, Italy, Turkey, and Jordan, ensuring proportional representation across Mediterranean and non-Mediterranean regions. To facilitate regional comparisons, countries were grouped as Mediterranean (MC)—including Tunisia, Algeria, Morocco, France, Spain, Italy, and Turkey—or non-Mediterranean (NMC)—including Germany, Luxembourg, and Jordan, based on strict geographic and historical definitions of the Mediterranean (26).

The survey was developed in multiple languages, including English, German, French, Italian, Spanish, Arabic, and Turkish, to maximize accessibility. Validated instruments were used to assess adherence to the Mediterranean diet and associated lifestyle factors. Items without official translations underwent rigorous translation and back-translation processes, ensuring high reliability coefficients (r = 0.81–0.94). The survey was hosted on the GDPR-compliant SoSci Survey platform, with support from Johannes Gutenberg University Mainz.

The MEDIET4ALL survey was disseminated through various channels, including email invitations, university and consortium websites, and social media platforms such as ResearchGate™, LinkedIn™, Facebook™, WhatsApp™, and X™ (formerly Twitter™). The MEDIET4ALL consortium and collaborators (e.g., Bilendi solution) facilitated distribution, and the general public was encouraged to share the survey within their networks.

Initially, more than 8,000 individuals participated in the survey. To ensure high-quality data, responses underwent logical screening to detect inconsistencies, such as reporting no vigorous physical activity while simultaneously claiming daily engagement in such exercises. Duplicate entries were identified and removed based on criteria including matching IP addresses, closely timed submissions, and highly similar demographic and response patterns. Implausible values, such as reporting unrealistic sleep durations (e.g., 24 h) or improbable dietary intake, were also excluded to enhance data reliability. After applying rigorous data filtering for accuracy and completeness, 4,010 valid responses were included in the final analysis.

The survey collected demographic, socio-economic, and behavioral data, including dietary behaviors, physical activity, social participation, sleep quality, mental health, and lifestyle characteristics. Several validated questionnaires were used, including the MedLife Index for dietary adherence (27), the International Physical Activity Questionnaire Short Form (IPAQ-SF) for physical activity (28), the Depression Anxiety Stress Scales-21 (DASS-21) for mental health assessment (29), and the Short Life Satisfaction Questionnaire (SLSQ) for well-being evaluation (30).

The survey began with an introductory page detailing the study’s objectives, ethical considerations, data privacy policies, and consent information. The participants were required to select one of the seven available languages before proceeding. The estimated completion time was 15–20 min. Participation was entirely voluntary, and no personal identifiable information was collected. Withdrawal was possible at any stage without any negative consequences.

This study involved human subjects, and all ethical guidelines, including informed consent, were strictly adhered to in accordance with the ethical principles of the Declaration of Helsinki. The protocol and consent form were approved by the Ethics Committee of the Faculty of Medicine at the University of Sfax (approval identification code: 066/24). Participation was entirely voluntary, and participants were informed that all data would be used solely for research purposes and kept anonymous and confidential. The survey complied with the SoSci Survey privacy policy,^1^ the Federal Data Protection Act (BDSG), and the EU General Data Protection Regulation (GDPR).

Participants were not asked to provide personally identifiable information, and their responses were recorded only upon survey submission. They were informed that they could withdraw from the study at any stage with no negative consequences. By completing the survey, participants provided informed consent for the anonymous use of their data for research purposes. Data confidentiality was rigorously maintained in compliance with applicable privacy regulations.

### 2.2 Measures and questionnaires

The survey used a variety of validated questionnaires to collect data on adherence to the MedDiet and associated lifestyle factors.

#### 2.2.1 MedLife Index

The MedDiet adherence scores were recently evaluated in a systematic review con-ducted by Zaragoza-Martí et al. (31). Among the 28 scores analyzed, 12 were applied to the general population, and five adhered to the principles of the MedDiet Pyramid (32, 33). However, only the “MedLife index,” developed by Sotos-Prieto et al. (27), demonstrated good internal consistency with a Cronbach’s *α* coefficient of 0.75. Based on these findings, the MedLife Index was selected for inclusion in the MedLife 4ALL e-survey.

The MedLife Index is a validated instrument that measures adherence to the MedDiet and associated lifestyle practices (27). Based on the MedDiet Pyramid (33), the index includes 28 items divided into three categories: food consumption frequency (15 items), dietary habits (7 items), and lifestyle behaviors (6 items). The first category, food consumption frequency, includes 15 items that evaluate adherence to MedDiet patterns, such as consuming adequate amounts of fruits, vegetables, whole grains, and healthy fats, while limiting the intake of pastries and red meat. The second category, MedDiet habits, consists of 7 items assessing behaviors such as minimizing salt and sugar use and avoiding snacking between meals. The third category, lifestyle behaviors, contains 6 items measuring physical activity (e.g., engaging in at least 150 min of moderate activity per week), sleep duration (6–8 h), social habits, and conviviality.

Each item in the index is scored as 0 for non-adherence or 1 for adherence, with the total score ranging from 0, representing the lowest adherence, to 28, indicating the highest adherence. To facilitate interpretation, scores are classified into three levels: low adherence for scores below 12, medium adherence for scores between 12 and 16, and high adherence for scores above 16. These thresholds are determined based on tertiles of the total score distribution within the dataset. The MedLife Index thus offers a comprehensive measure of adherence to the MedDiet and lifestyle model.

#### 2.2.2 Four items from the Pittsburgh Sleep Quality Index

Four items from the Pittsburgh Sleep Quality Index (PSQI) were included in the survey to assess sleep quality and disturbances (34), focusing on sleep efficiency, sleep latency, sleep quality, and sleep du-ration. Sleep efficiency was defined as the ratio of time asleep to time in bed, categorized as good when exceeding 85% or poor when below 85%. Sleep latency referred to the time taken to fall asleep, classified as good if less than 20 min or delayed if greater than 20 min. Sleep quality was self-rated on a 4-point Likert scale ranging from “very good” to “very bad.” Sleep duration was evaluated based on age-specific recommendations, with 7 to 9 h considered adequate for individuals younger than 65 years and 7 to 8 h for those aged 65 or older (35). The total PSQI score ranged from 0 to 21, with higher scores indicating poorer sleep quality.

#### 2.2.3 Insomnia Severity Index

The Insomnia Severity Index (ISI) is a self-reported questionnaire designed to assess the severity and impact of insomnia symptoms (36). It includes seven items that evaluate difficulties with falling asleep, staying asleep, waking too early, satisfaction with sleep, interference with daily functioning, noticeability of sleep problems, and the personal distress caused by these difficulties. Each item is rated on a 0–4 scale, producing a total score range of 0 to 28. Based on the total score, insomnia is categorized into four levels: absence of insomnia (0–7), sub-threshold insomnia (8–14), moderate insomnia (15–21), or severe insomnia (22–28). The ISI is widely recognized for its reliability and ease of use, making it a valuable tool for clinical assessments, research, and monitoring treatment outcomes (36).

#### 2.2.4 Depression Anxiety Stress Scales-21

The Depression Anxiety Stress Scales-21 (DASS-21) is a validated self-report questionnaire designed to measure the se-verity of symptoms related to depression, anxiety, and stress over the past week. It consists of 21 items, with seven items allocated to each of the three scales. Responses are rated on a 4-point Likert scale, and the scores for each scale are summed and then multiplied by two to reflect the overall severity. The scores categorize symptoms into five levels: normal, mild, moderate, severe, and extremely severe, offering valuable in-sights for clinical assessment and research into mental health (37).

#### 2.2.5 Short Life Satisfaction Questionnaire-lockdown

The Short Life Satisfaction Questionnaire (SLSQ) is a modified version of the Satisfaction with Life Scale (SWLS) that focuses on three items closely associated with emotional well-being. Validated during the COVID-19 lockdown period (38, 39). The SLSQ allows participants to evaluate their life satisfaction based on personal criteria. Participants rate their agreement with each item on a 7-point Likert scale, ranging from 1 (“Strongly disagree”) to 7 (“Strongly agree”), producing a total score between 3 and 21. Higher scores indicate greater life satisfaction and are categorized as follows: 3 (“Extremely dissatisfied”), 4–6 (“Dissatisfied”), 7–9 (“Slightly dissatisfied”), 10–12 (“Neutral”), 13–15 (“Slightly satisfied”), 16–18 (“Satisfied”), and 19–21 (“Extremely satisfied”).

#### 2.2.6 International Physical Activity Questionnaire Short Form

The International Physical Activity Questionnaire Short Form (IPAQ-SF) is a self-reported tool designed to quantify physical activity levels across various intensities, including vigorous, moderate, and walking activities, over the past 7 days. It calculates the total activity in MET-minutes per week (Metabolic Equivalent Task), where activities are classified into three levels: low activity (<1,500 MET-minutes/week), moderate activity (1,500–2,999 MET-minutes/week), and high activity (≥3,000 MET-minutes/week). The IPAQ-SF is widely used for evaluating physical activity patterns in diverse populations and supports both clinical assessments and research studies on physical activity and health outcomes (40, 41).

#### 2.2.7 Short Social Participation Questionnaire-Lockdowns

The Short Social Participation Questionnaire (SSPQ) is a short modified version of the Social Participation Questionnaire (SPQ) designed to assess social participation behaviors during the last 12 months and previously validated and used during the COVID-19 home confinement period (38, 39). It includes 14 items, with 10 items rated on a 5-point scale from “never” to “all the time,” and 4 items requiring binary “yes” or “no” responses. The total score ranges from 14 to 70, with higher scores indicating greater levels of social participation. Scores are categorized as follows: 14 (“Never socially active”), 15–28 (“Rarely socially active”), 29–42 (“Sometimes socially active”), 43–56 (“Often socially active”), and 57–70 (“Socially active at all times”).

#### 2.2.8 Short Technology-Use Questionnaire-Lockdowns

The Short Technology-Use Questionnaire-Lockdowns (STuQL) was developed to assess technology use for social participation, dietary practices, and physical activity. The questionnaire was previously validated and used during the COVID-19 home confinement period (38, 39). The questionnaire consists of three items rated on a 5-point scale ranging from “never” to “all the time.” Scores range from 3, indicating minimal technology use, to 15, indicating extensive use, with intermediate scores reflecting varying levels of engagement with technology.

#### 2.2.9 The MedDiet Barriers Questionnaire

The MedDiet Barriers Questionnaire (MBQ) is a newly developed tool designed to assess barriers and limitations to adherence to the MedDiet. The questionnaire includes 13 items exploring the presence (answered as “Yes”) or absence (answered as “No”) of various potential barriers identified in the literature. Specifically, the MBQ addresses: (i) barriers related to food allergies and intolerances; (ii) barriers related to cultural and/or religious limitations; (iii) barriers related to medical or health-related limitations; (iv) barriers related to individual beliefs (e.g., vegan or vegetarian diets); (v) barriers related to taste dislike; (vi) barriers related to attitudes, such as suitability, taste, restrictiveness, or food waste concerns; (vii) barriers related to social norms (e.g., food culture); (viii) barriers related to low motivation; (ix) barriers related to price unaffordability; (x) barriers related to time or effort-consuming meal preparation; (xi) barriers related to low accessibility or availability of Mediterranean food; (xii) barriers related to lack of knowledge and cooking skills; and (xiii) other barriers. Responses are scored as “No” = 0 and “Yes” = 1, with the total score calculated as the sum of all items, ranging from 0 to 13. A score of 0 indicates the absence of barriers, while a score of 13 reflects severe barriers to adherence.

#### 2.2.10 Additional data

Additional geo-demographic, socio-economic, and health-related data were collected, along with information on participants’ awareness of the MedDiet. Demographic variables included age, gender, marital status, education, employment, living environment, and ethnicity. Health status was categorized into healthy, at risk of disease, or living with diseases.

### 2.3 Statistical analysis

All analyses were performed using SPSS version 25. Descriptive statistics summarized the distribution of MedLife adherence categories across MC and NMC regions, with results expressed as respondents number as well as percentages of respondents within each region. A chi-square test of independence (χ^2^) was performed to assess the overall associations between regional and each categorical variable. Then, to assess whether the proportion of participants in each categorical variable differed significantly between regions, a Z-test for two proportions was conducted using the following pooled variance formula:


$$z=\frac{{\hat{p}}_{1}-{\hat{p}}_{2}}{\sqrt{\hat{p}\left(1-\hat{p}\right)\left(\frac{1}{n_{1}}+\frac{1}{n_{2}}\right)}}$$

where:

- ${\hat{p}}_{1}\;$and ${\hat{p}}_{2}\;$are the observed proportions of a given category in the NMC and MC groups, respectively;
- $n_{1}$ (NMC: 1416) and $n_{2}$ (MC: 2594) represent the sample sizes for each region;
- $\hat{p}\;$is the pooled proportion, calculated as $\frac{x1+x2}{n1+n2}\;$where x1 and x2are the counts of participants in the category of interest for NMC and MC.

This test accounts for the imbalanced sample sizes between regions, with significance thresholds set at ∣Z∣ ≥ 1.96 (*p* < 0.05), ∣Z∣ ≥ 2.58 (*p* < 0.01), and ∣Z∣ ≥ 3.29 (*p* < 0.001). For continuous variables, the Shapiro–Wilk test confirmed non-normality, prompting the use of Mann–Whitney U tests. Effect sizes were reported using Cohen’s h for proportional differences (interpreted as: trivial ≤ 0.20; small > 0.20; medium > 0.50; large > 0.80) and Cohen’s d for mean comparisons, per Hopkins’ criteria. All tests were two-tailed with *α* = 0.05.

## 3 Results

### 3.1 Demographic characteristics of the participants

The demographic characteristics of the participants are presented in Table 1. Of the 4,010 participants, 2,594 (64.7%) resided in Mediterranean countries (MC), while 1,416 (35.3%) were from non-Mediterranean countries (NMC). Among NMC participants, 682 (48.2%) were from Asia, whereas MC participants were predominantly from Europe (*n* = 1,552, 58.7%) and Africa (*n* = 476, 18.4%) (*p* < 0.001).

**Table 1:** Sociodemographic characteristics of the study population (*n* = 4,010) stratified by regions.

Variables	NMC	MC	Total	Region effect		
				χ^2^	df	*p*
Country of living	1,416	2,594	4,010	4,010	9	<0.001
Algeria			146			
France			533			
Germany			616			
Italy			711			
Luxembourg			118			
Tunisia			170			
Spain			278			
Morocco			160			
Turkey			596			
Jordan			682			
Continent						
Europe	734 (51.8%)	1,522 (58.7%)^***^	2,256	449.78	2	<0.001
Asia	682 (48.2%)	596 (23%)^***^	1,278			
Africa	0 (0%)	476 (18.4%)^***^	476			
Ethnicity						
Prefer not to say	2 (0.1%)	199 (7.7%)^***^	201	849.24	7	<0.001
Black/African/Caribbean	10 (0.7%)	115 (4.4%)^***^	125			
Latin American/Hispanic	1 (0.1%)	61 (2.4%)^***^	62			
White/European	671 (47.4%)	1,269 (48.9%)	1940			
Asian	50 (3.5%)	60 (2.3%)^*^	110			
Middle Eastern / Arab	620 (43.8%)	286 (11%)^***^	906			
Turks	23 (1.6%)	527 (20.3%)^***^	550			
Other	39 (2.8%)	77 (3%)	116			
Living environment						
Urban environment	919 (64.9%)	1739 (67%)	2,658	2.733	2	0.255
Suburban environment	275 (19.4%)	451 (17.4%)	726			
Rural environment	222 (15.7%)	404 (15.6%)	626			
Age (years)						
18–35	887 (62.6%)	1,283 (49.5%)^***^	2,170	64.69.2	2	<0.001
36–55	329 (23.2%)	842 (32.5%)^***^	1,171			
>55	200 (14.1%)	469 (18.1%)^***^	669			
Gender						
Male	429 (30.3%)	1,196 (46.1%)^***^	1,625	94.99	1	<0.001
Female	987 (69.7%)	1,398 (53.9%)^**^	2,385			
BMI						
Underweight	77 (5.4%)	132 (5.1%)	209	9.65	3	0.022
Normal weight	717 (50.6%)	1,429 (55.1%)^**^	2,146			
Overweight	602 (42.5%)	1,012 (39%)^*^	1,614			
Obesity	20 (1.4%)	21 (0.8%)	41			
Education						
No schooling completed	38 (2.7%)	170 (6.6%)^***^	208	144.36	3	<0.001
High school graduate, diploma or the equivalent/Professional degree	494 (34.9%)	950 (36.6%)	1,444			
Bachelor’s degree	665 (47%)	790 (30.5%)^***^	1,455			
Master-doctorate degree	219 (15.5%)	684 (26.4%)^***^	903			
Marital status						
Single	840 (59.3%)	1,133 (43.7%)^***^	1973	103.18	2	<0.001
Married living as couple	466 (32.9%)	1,280 (49.3%)^***^	1746			
Widowed, Divorced, Separated	110 (7.8%)	181 (7%)	291			
Employment						
Employed	639 (45.1%)	1,394 (53.7%)^***^	2033	89.95	4	<0.001
Unemployed	218 (15.4%)	249 (9.6%)^***^	467			
Student	440 (31.1%)	587 (22.6%)^***^	1,027			
Retired	87 (6.1%)	252 (9.7%)^***^	339			
Uncategorized	32 (2.3%)	112 (4.3%)^***^	144			
Health status						
Healthy	1,039 (73.4%)	1975 (76.1%)	3,014	3.83	2	0.148
At risk	266 (18.8%)	442 (17%)	708			
With diseases	111 (7.8%)	177 (6.8%)	288			
Smoking						
Cigarettes smokers	203 (14.3%)	592 (22.8%)^***^	795	41.68	2	<0.001
Shisha smokers	76 (5.4%)	133 (5.1%)	209			
Non-smokers	1,137 (80.3%)	1869 (72.1%)^***^	3,006			

^*^Significant difference compared to NMC at *p* < 0.05; ^**^significant difference compared to NMC at *p* < 0.01; ^***^significant difference compared to NMC at *p* < 0.001.

Ethnic background varied significantly across regions (*p* < 0.001), with only the white/European ethnic group being the largest group in both MC and NMC, showing comparable proportions (≈50%). Middle Eastern/Arab participants (43.8%) were significantly more represented in the NMC (*p* < 0.001), while Turkish participants (20.3%) were significantly more represented in the MC (*p* < 0.001).

The age and gender distribution revealed significant regional differences (*p* < 0.001), with younger individuals (18–35 years) and females representing the largest categories in both regions. However, the proportions were higher in the NMC compared to the MC (62.6% vs. 49.5% for younger individuals and 69.7% vs. 53.9% for females) (*p* < 0.001). BMI classification showed significant variation (*p* = 0.022), with normal weight being the most common category in both regions, where MC participants had a higher prevalence of normal weight (55.1% vs. 50.6%; *p* < 0.01) and a lower prevalence of overweight individuals (39% vs. 42.5%; *p* < 0.05).

Educational attainment differed significantly across regions (*p* < 0.001), with individuals holding bachelor’s degrees being more prominent in the NMC, showing a higher proportion than in the MC (47% vs. 30.5%). Conversely, a higher proportion of individuals with a master’s or doctorate degree was found in the MC (26.4% vs. 15.5%). Marital status also varied significantly (*p* < 0.001), with a greater proportion of single individuals in the NMC (59.3% vs. 51.5%) and more married or cohabiting individuals in the MC (49.3% vs. 32.9%; *p* < 0.001).

Employment status showed significant regional differences (*p* < 0.001). MC participants had higher employment (53.7% vs. 45.1%; *p* < 0.001) and retirement (9.7% vs. 6.1%; *p* < 0.001) rates, whereas NMC participants had a greater proportion of unemployed (15.4% vs. 9.6%; *p* < 0.001) and students (31.1% vs. 22.6%; *p* < 0.001) participants. Smoking behavior exhibited notable regional disparities (p < 0.001), with non-smokers presented higher proportion in NMC (80.3% vs. 72.1%; *p* < 0.001).

Living environment and health status showed comparable subgroup prevalences between MC and NMC, with no significant differences (*p* > 0.05).

### 3.2 Observational results for all parameters

#### 3.2.1 MedLife Index and perceived barriers

The Mann–Whitney test revealed significant differences in total MedLife Index scores across regions (Z = 5.03, *p* < 0.001, Cohen’s d = 0.17), with MC participants scoring higher (mean = 14.2 ± 3.18) than NMC participants (mean = 13.66 ± 3.18) (Table 2).

**Table 2:** Analysis of the MedLife Index: Mediterranean diet adherence, dietary habits, and consumption patterns by regions.

MedLife index		*n*			Region effect		
Food group	Criteria for 1 point	NMC	MC	Total			
					*χ^2^*	*df*	*p*
Block 1: Mediterranean food consumption							
Sweets	≤2 servings/week	688 (48.6%)	1,573 (60.6%)^***^	2,261	54.1	1	<0.001
Red meat	<2 servings/week	795 (56.1%)	1,192 (46%)^***^	1987	38.06	1	<0.001
Processed meat	≤1 serving/week	845 (59.7%)	1,426 (55%)^**^	2,271	8.25	1	0.004
Eggs	2–4 servings/week	601 (42.4%)	1,225 (47.2%)^*^	1826	8.44	1	0.004
Legumes	≥2 servings/week	749 (52.9%)	1,681 (64.8%)^***^	2,430	54.4	1	<0.001
White meat	2 servings/week	250 (17.7%)	684 (26.4%)^***^	934	38.92	1	<0.001
Fish/seafood	≥2 servings/week	279 (19.7%)	1,023 (39.4%)^***^	1,302	162.68	1	<0.001
Potatoes	≤3 servings/week	1,193 (84.3%)	2,164 (83.4%)	3,357	0.46	1	0.497
Low-fat dairy products	2 servings/d	199 (14.1%)	383 (14.8%)	582	0.373	1	0.541
Nuts and olives	1–2 servings/d	651 (46%)	1,174 (45.3%)	2,185	0.19	1	0.663
Herbs, spices and garnish	≥1 serving/d	1,313 (92.7%)	2,333 (89.9%)^**^	3,646	8.625	1	0.003
Fruit	3–6 servings/d	229 (16.2%)	464 (17.9%)	693	1.89	1	0.17
Vegetables	≥2 servings/d	693 (48.9%)	1,303 (50.2%)	1996	0.61	1	0.435
Olive oil	≥3 servings/d	437 (30.9%)	782 (30.1%)	1,219	0.22	1	0.638
Cereals	3–6 servings/d	439 (31%)	542 (20.9%)^***^	981	50.65	1	<0.001
*Block 1: Total score*					*Z*	*p*	*Cohen’s d*
		6.69 ± 1.94	7.01 ± 1.94		−4.71	<0.001	0.16
Block 2: Mediterranean dietary habits							
Water or infusions	6–8 servings/d or ≥3 servings/week	1,062 (75%)	2,105 (81.1%)^***^	3,167	20.86	1	<0.001
Wine	1–2 servings/d	75 (5.3%)	275 (10.6%)_***_	350	32.36	1	<0.001
Limit salt in meals	Yes	785 (55.4%)	1758 (67.8%)^***^	2,543	60.06	1	<0.001
Preference for whole grain products	Yes/fiber > 25 g/d	956 (67.5%)	1,434 (55.3%)^***^	2,390	56.93	1	<0.001
Snacks	≤2 servings/week	928 (65.5%)	1943 (74.9%)^***^	2,871	39.52	1	<0.001
Limit nibbling between meals	Yes	872 (61.6%)	1,685 (65%)^*^	2,557	4.52	1	0.034
Limit sugar in beverages (including sugar-sweetened beverages)	Yes	1,049 (74.1%)	1906 (73.5%)	2,955	0.173	1	0.678
*Block 2: Total score*					*Z*	*p*	*Cohen’s d*
		4.04 ± 1.61	4.28 ± 1.51		4.46	<0.001	0.15
Block 3: Physical activity, rest, social habits and conviviality							
Physical activity (>150 min/week or 30 min/d)	Yes	840 (59.3%)	1,579 (60.9%)	2,419	0.919	1	0.338
Siesta/nap	Yes	643 (45.4%)	940 (36.2%)^***^	1,583	32.25	1	<0.001
Hours of sleep	6–8 h/d	983 (69.4%)	1858 (71.6%)	2,841	2.16	1	0.142
Watching television	<1 h/d	328 (23.2%)	492 (19%)^**^	820	9.92	1	0.002
Socializing with friends	≥2 h/weekend	984 (69.5%)	1908 (73.6%)^**^	2,892	7.52	1	0.006
Collective sports	≥2 h/week	363 (25.6%)	768 (29.6%)^**^	1,131	7.13	1	0.008
*Block 3: Total score*					*Z*	*p*	*Cohen’s d*
		2.92 ± 1.28	2.91 ± 1.26		−0.777	0.437	0.01
MedLife categories							
Low		491 (34.7%)	757 (29.2%)^***^	1,248	17.82	2	<0.001
Medium		654 (46.2%)	1,221 (47.1%)	1875			
High		271 (19.1%)	616 (23.7%) ***	887			
*Medlife index*					*Z*	*p*	*Cohen’s d*
		13.66 ± 3.18	14.2 ± 3.12		5.03	<0.001	0.17

^*^Significant difference compared to NMC at *p* < 0.05; ^**^significant difference compared to NMC at *p* < 0.01; ^***^significant difference compared to NMC at *p* < 0.001.

Participants in MC presented significantly higher scores in Block 1 (Mediterranean Food Consumption) (Z = −4.71, *p* < 0.001, Cohen’s d = 0.16), with greater adherence to recommended intake in key food groups: sweets (60.6% vs. 48.6%; *p* < 0.001), eggs (47.2% vs. 42.4%; *p* < 0.05), legumes (64.8% vs. 52.9%; *p* < 0.001), white meat (26.4% vs. 17.7%; *p* < 0.001), and fish/seafood (39.4% vs. 19.7%, *p* < 0.001). However, NMC reported higher adherence to recommended intake of red meat (56.1% vs. 46%; *p* < 0.001), processed meat (59.7% vs. 55%; *p* < 0.01), herbs, spices and garnish (92.7% vs. 89.9%; *p* < 0.01), and cereals (31% vs. 20.9%; *p* < 0.001).

Block 2 (Mediterranean Dietary Habits) showed significant regional differences (Z = 4.46, *p* < 0.001, Cohen’s d = 0.15) with higher scores in MC. MC participants presented higher proportions in water (81.1% vs. 75%; *p* < 0.001), wine (10.6% vs. 5.3%; *p* < 0.001), limited salt in meals (67.8% vs. 55.4%; *p* < 0.001), limited snacks (74.9% vs. 65.5%; *p* < 0.001) and limited nibbling between meals (65% vs. 61.6%; *p* < 0.05), while NMC participants reported stronger preference for whole grains (67.5% vs. 55.3% *p* < 0.001).

Block 3 (physical activity, rest, social habits, and conviviality) score did not significantly differ between MC and NMC (Z = −0.777, *p* = 0.437, Cohen’s d = 0.01). Looking at the individual items, the Z-test for two proportions showed significantly greater participation in socializing with friends and engaging in collective sports practices for MC participants (73.6% vs. 69.5% and 29.6% vs. 25.6%; *p* < 0.01 for both), while NMC participants demonstrated significantly greater participation in napping (45.4% vs. 36.2%; *p* < 0.001) and limiting television watching (23.2% vs. 19%; *p* < 0.01). Regarding the MedLife Index categories (low, medium, and high adherence), MC participants reported a lower proportion in the low adherence category (29.2% vs. 34.7%; *p* < 0.001) and a higher proportion in the high adherence category (23.7% vs. 19.1%; *p* < 0.001).

For perceived barriers, there was no significant difference between MC and NMC (Z = −0.93, *p* = 0.356, Cohen’s d = 0.06). However, the Z-test for two proportions revealed a higher proportion of barriers related to awareness (59.8% vs. 44.8%; *p* < 0.001), attitudes (83% vs. 79.7%; *p* < 0.05), price unaffordability (72.9% vs. 69.6%; *p* < 0.05), low accessibility (87.4% vs. 84.3%; *p* < 0.01), and individual beliefs (91.6% vs. 89.7%; *p* < 0.05) among MC participants. In contrast, a higher proportion of NMC participants reported barriers related to low motivation (19.3% vs. 16.4%; *p* < 0.05), time effort consumption (28.3% vs. 22.1%; *p* < 0.001), and lack of knowledge (20% vs. 17%; *p* < 0.05) (Table 3).

**Table 3:** Regions differences in awareness and potential barriers influencing their adherence to MedLife.

	Yes: *n* (%)			Region effect		
Variables	NMC	MC	Total	*χ^2^*	*df*	*p*
Awareness	634 (44.8%)	1,551 (59.8%)^***^	2,185	83.31	1	<0.001
Attitudes	1,129 (79.7%)	2,153 (83%)^*^	3,282	6.58	1	0.01
Social norms	1,168 (82.5%)	2,164 (83.4%)	3,332	0.57	1	0.449
Low motivation	273 (19.3%)	426 (16.4%)^*^	699	4.19	1	0.023
Price unaffordability	986 (69.6%)	1891 (72.9%)^*^	2,877	4.82	1	0.028
Time/ effort consuming	401 (28.3%)	573 (22.1%)^***^	974	19.33	1	<0.001
Low accessibility	1,193 (84.3%)	2,266 (87.4%)^**^	3,459	7.45	1	0.006
Lack of knowledge	283 (20%)	439 (16.9%)^*^	722	5.82	1	0.016
Food allergies and intolerances	197 (13.9%)	343 (13.2%)	540	0.37	1	0.541
Cultural and/or religious reason	1,284 (90.7%)	23,025 (887.6%)	3,589	3.23	1	0.072
Medical reason	1,292 (91.2%)	2,303 (88.8%)^*^	3,595	5.98	1	0.014
Individual beliefs (e.g., vegan, vegetarian)	1,270 (89.7%)	2,377 (91.6%)^*^	3,647	4.21	1	0.04
Taste dislike	275 (19.4%)	466 (18%)	741	1.29	1	0.256
Others	37 (2.6%)	50 (1.9%)	87	2.03	1	0.154
*Total score*				*Z*	*p*	*Cohen’s d*
	6.91 ± 1.29	6.85 ± 1.28		−0.932	0.356	0.06

^*^Significant difference compared to NMC at *p* < 0.05; ^**^significant difference compared to NMC at *p* < 0.01; ^***^significant difference compared to NMC at *p* < 0.001.

#### 3.2.2 Physical and social activities, sleep patterns, and technology use behavior

There was no significant difference between regions in overall physical activity behavior (Z = 0.31, *p* = 0.758, Cohen’s d = 0.01), with the majority of participants from both regions (60–62%) primarily engaging in low physical activity. However, the Z-test for two proportions showed a higher proportion of participants engaging in moderate activities in MC compared to NMC (21.1% vs. 18.5%; *p* < 0.05). Regarding social participation behaviors, the majority of participants from both regions were classified as “sometimes socially active,” with a higher proportion in MC (52.2%) compared to NMC (48.1%). In contrast, NMC showed a significantly higher proportion, albeit small, of individuals categorized as “always socially active” (5.9%) compared to MC (3.2%). Overall, the SSPQ score was slightly higher in NMC compared to MC (Z = −2.48, *p* = 0.013, Cohen’s d = 0.1) (Table 4).

**Table 4:** Behavioral and sleep outcomes by regions.

Variables	*n*			Region effect		
	NMC	MC	Total	*χ^2^*	*df*	*p*
IPAQ-SF score						
Low activities	875 (61.8%)	1,543 (59.5%)	2,418	3.97	2	0.137
Moderate activities	262 (18.5%)	548 (21.1%)^*^	810			
High activities	279 (19.7%)	503 (19.4%)	782			
*IPAQ-SF score*				*Z*	*p*	*Cohen’s d*
	1759.07 ± 2216.5	1772.58 ± 2155.63		0.31	0.758	0.01
SSPQ score						
Never	10 (0.7%)	10 (0.4%)	20	22.97	4	<0.001
Rarely	245 (17.3%)	464 (17.9%)	709			
Sometimes	681 (48.1%)	1,354 (52.2%)^*^	2035			
Often	369 (26.1%)	684 (26.4%)	1,080			
All times	84 (5.9%)	82 (3.2%)^***^	166			
*SSPQ*: *Total score*				*Z*	*p*	*Cohen’s d*
	38.55 ± 10.66	37.55 ± 9.71		−2.48	0.013	0.1
Sleep efficiency						
>85	1,271 (89.8%)	2,297 (88.6%)	3,568	1.36	1	0.242
<85	145 (10.2%)	297 (11.4%)	442			
*Sleep efficiency*				*Z*	*p*	*Cohen’s d*
	93.31 ± 6.75	93.05 ± 7.37		0.34	0.733	0.04
Sleep latency						
<20	816 (57.6%)	1,517 (58.5%)	2,333	0.275	1	0.6
>20	600 (42.4%)	1,077 (41.5%)	1,677			
*Sleep latency*				*Z*	*p*	*Cohen’s d*
	30.14 ± 32.17	30.55 ± 34.49		−1.22	0.224	0.01
Sleep quality						
Very bad	80 (5.6%)	143 (5.5%)	223	22.5	3	<0.001
Fairly bad	298 (21%)	704 (27.1%)^***^	1,002			
Fairly good	749 (52.9%)	1,321 (50.9%)	2070			
Very good	289 (20.4%)	426 (16.4%)^**^	715			
*Sleep quality*				*Z*	*p*	*Cohen’s d*
	2.88 ± 0.79	2.78 ± 0.78		−4.17	<0.001	0.13
Sleep duration						
Below recommendation	565 (39.9%)	1,165 (44.9%)^**^	1730	20.72	2	<0.001
Within recommendation	737 (52%)	1,301 (50.2%)	2038			
Exceeded recommendation	114 (8.1%)	128 (4.9%)^***^	242			
*Sleep duration*				*Z*	*p*	*Cohen’s d*
	6.99 ± 1.79	6.73 ± 1.64		−4.24	<0.001	0.15
Insomnia						
Absence of insomnia	512 (36.2%)	1,026 (39.6%)^*^	1,538	19.48	3	<0.001
Sub-threshold insomnia	549 (38.8%)	1,067 (41.1%)	1,616			
Moderate insomnia	302 (21.3%)	439 (16.9%)^***^	741			
Severe insomnia	53 (3.7%)	62 (2.4%)^*^	115			
*ISI: Total score*				*Z*	*p*	*Cohen’s d*
	10.29 ± 5.86	9.32 ± 5.78		−4.89	<0.001	0.17
Technology use behavior						
Rarely	49 (3.5%)	125 (4.8%)^*^	174	5.57	3	0.135
Sometimes	212 (15%)	382 (14.7%)	594			
Often	563 (39.8%)	1,064 (41%)	1,627			
All times	592 (41.8%)	1,023 (39.4%)	1,615			
*Technology use behavior*				*Z*	*p*	*Cohen’s d*
	8.98 ± 2.72	8.85 ± 2.75		−1.33	0.184	0.05

^*^Significant difference compared to NMC at *p* < 0.05; ^**^significant difference compared to NMC at *p* < 0.01; ^***^significant difference compared to NMC at *p* < 0.001. IPAQ-SF, The International Physical Activity Questionnaires-short form; SSPQ-L, Short Social Participation Questionnaire. ISI, Insomnia Severity Index.

Regarding sleep patterns, no significant differences were found in sleep efficiency (Z = 0.34, *p* = 0.733, Cohen’s d = 0.04) and sleep latency (Z = −1.22, *p* = 0.224, Cohen’s d = 0.01). However, sleep quality was significantly lower in MC (Z = −4.17, *p* < 0.001, Cohen’s d = 0.13), with MC participants reporting higher proportions of “Fairly bad” sleep quality (27.1% vs. 21%; *p* < 0.001) and lower proportions of “Very good” sleep quality (16.4% vs. 20.4%; *p* < 0.01) compared to NMC. Additionally, MC had significantly lower sleep duration (Z = −4.24; *p* < 0.001; Cohen’s d = 0.15), with a higher proportion of participants reporting sleep durations below the recommended amount (44.9% vs. 39.9%; *p* < 0.01) and a lower proportion exceeding the recommended sleep duration (4.9% vs. 8.1%; *p* < 0.001). Regarding insomnia, MC had a lower insomnia score (Z = −4.89; *p* < 0.001; Cohen’s d = 0.17), with a greater proportion reporting no insomnia (39.6% vs. 36.2%; *p* < 0.05). In contrast, NMC participants showed higher proportions of moderate and severe insomnia (21.3% vs. 16.9% and 3.7% vs. 2.4%; *p* < 0.001 and *p* < 0.05, respectively).

There was no significant regional effect on technology use behavior scores (Z = −1.33, *p* = 0.184, Cohen’s d = 0.01), with the majority of participants from both regions reporting frequent (often) to constant (all times) technology use (≈80%).

#### 3.2.3 Life satisfaction, mental health (DASS-21), and the need for psychosocial, physical, and nutritional support

There were no significant regional differences in terms of life satisfaction, depression, anxiety, and stress (*p* > 0.05). However, it should be noted that the highest proportion in both regions were declaring moderate depression and anxiety (33–35%) (Table 5).

**Table 5:** Assessment of life satisfaction, mental health (DASS-21), and the demand for psychosocial, physical, and nutritional support.

Variables	*n*			Region effect		
	NMC	MC	Total	*χ^2^*	*df*	*p*
*Life satisfaction questionnaire*						
Extremely dissatisfied	31 (2.2%)	68 (2.6%)	99	5.17	6	0.522
Dissatisfied	60 (4.2%)	106 (4.1%)	166			
Slightly dissatisfied	120 (8.5%)	248 (9.6%)	368			
Neutral	344 (24.3%)	642 (24.7%)	986			
Slightly satisfied	315 (22.2%)	579 (22.3%)	894			
Satisfied	315 (22.2%)	579 (22.3%)	825			
Extremely satisfied	259 (18.3%)	413 (15.9%)	672			
*Life satisfaction questionnaire score*				*Z*	*p*	*Cohen’s d*
	14.41 ± 4.51	14.15 ± 4.52		−1.76	0.078	0.16
DASS 21						
*Depression*						
Normal	390 (27.5%)	713 (27.5%)	1,103	1.198	4	0.878
Mild	396 (28%)	723 (27.9%)	1,119			
Moderate	487 (34.4%)	900 (34.7%)	1,387			
Severe	135 (9.5%)	236 (9.1%)	371			
Extremely severe	8 (0.6%)	22 (0.8%)	30			
*Depression score*				*Z*	*p*	*Cohen’s d*
	13.37 ± 5.06	13.36 ± 4.68		0.09	0.924	0
*Anxiety*						
Normal	241 (17%)	400 (15.4%)	641	3.76	4	0.44
Mild	297 (21%)	542 (20.9%)	839			
Moderate	466 (32.9%)	924 (35.6%)	1,390			
Severe	272 (19.2%)	482 (18.6%)	754			
Extremely severe	140 (9.9%)	246 (9.5%)	386			
*Anxiety score*				*Z*	*p*	*Cohen’s d*
	12.36 ± 4.82	12.32 ± 4.68		0.159	0.874	0.01
*Stress*						
Normal	839 (59.3%)	1,487 (57.3%)	2,326	3.76	3	0.288
Mild	306 (21.6%)	628 (24.2%)	934			
Moderate	224 (15.8%)	437 (16.8%)	681			
Severe	27 (1.9%)	42 (1.6%)	69			
Extremely severe	0 (0%)	0 (0%)	0			
*Stress score*				*Z*	*p*	*Cohen’s d*
	14.07 ± 4.87	14.21 ± 4.72		1.15	0.249	0.03
Need of support						
*Psycho-social support*						
Never	370 (26.1%)	823 (31.7%)^***^	1,193	24.21	4	<0.001
Rarely	333 (23.5%)	664 (25.6%)	997			
Sometimes	485 (34.3%)	773 (29.8%)^**^	1,258			
Often	162 (11.4%)	245 (9.4%)^*^	407			
All times	66 (4.7%)	89 (3.4%)	155			
*Psycho-social support score*				Z	*p*	*Cohen’s d*
	2.45 ± 1.13	2.27 ± 1.11		−4.84	<0.001	0.16
*Physical support*						
Never	383 (27%)	746 (28.8%)	1,129	12.94	4	0.012
Rarely	244 (17.2%)	515 (19.9%)^*^	759			
Sometimes	446 (31.5%)	789 (30.4%)	1,235			
Often	230 (16.2%)	398 (15.3%)	628			
All times	113 (8%)	146 (5.6%)^**^	259			
*Physical support score*				*Z*	*p*	*Cohen’s d*
	2.61 ± 1.26	2.49 ± 1.21		−2.7	0.007	0.01
*Nutritional support*						
Never	365 (25.8%)	716 (27.6%)	1,081	3.88	4	0.423
Rarely	295 (20.8%)	514 (19.8%)	809			
Sometimes	412 (29.1%)	768 (29.6%)	1,180			
Often	215 (15.2%)	396 (15.3%)	611			
All times	129 (9.1%)	200 (7.7%)	329			
*Nutritional support score*				*Z*	*p*	*Cohen’s d*
	2.61 ± 1.27	2.56 ± 1.25		−1.18	0.24	0.04

^*^Significant difference compared to NMC at *p* < 0.05; ^**^significant difference compared to NMC at *p* < 0.01; ^***^significant difference compared to NMC at *p* < 0.001.

Regarding the need for support, NMC participants reported a significantly greater demand for psychosocial (Z = −4.84, *p* < 0.001, Cohen’s d = 0.16) and physical (Z = −2.7 *p* = 0.007, Cohen’s d = 0.01) supports. In psychosocial support NMC reported a significantly lower proportion for “Never” (26.1%vs. 31.7%; *p* < 0.001) and significantly higher proportion for “Sometimes” (34.3% vs. 29.8%; *p* < 0.01) and “Often” (11.4% vs. 9.4%; *p* < 0.05). Similarly for physical support, NMC presented significantly lower significant proportions for “Rarely” (17.2%vs. 19.9%; *p* < 0.05) and higher proportion for “All times” (8% vs. 5.6%; *p* < 0.01).

## 4 Discussion

The present study aims to evaluate regional variations in MedDiet adherence by analyzing socio-demographic and lifestyle factors across MC and NMC regions. The main findings highlight regional differences in adherence to the MedDiet and its associations with physical and mental health across MC and NMC regions. While MC participants showed greater adherence to traditional MedDiet components such as legumes and fish, NMC participants focused more on whole grains and modern adaptations of the diet. Distinct yet overlapping barriers to MedDiet adherence were identified across regions, with MC participants faced economic and accessibility challenges, while NMC participants struggled with knowledge gaps, behavioral hurdles, and time constraints. No significant differences in overall physical activity were observed, although MC participants engaged more in moderate physical activities and social participation. Sleep patterns varied, with MC participants reporting poorer sleep quality and shorter sleep duration compared to NMC. Mental health symptoms, particularly depression and anxiety, were moderate in both regions, likely reflecting global trends in mental health following the COVID-19 pandemic.

### 4.1 Demographic characteristics

The demographic differences observed between the MC and NMC populations are consistent with established regional patterns in various studies. Our findings reveal significant differences in age distribution, gender, BMI, educational attainment, marital status, and employment patterns between the two regions. These differences align with previous research, including that of Obeid et al. (26), who observed distinct demographic trends between Mediterranean and non-Mediterranean regions, including variations in education levels and employment patterns. The greater representation of MC participants from Europe and Africa, compared to the predominantly Asian background of NMC participants, reflects both geographic proximity to the Mediterranean Sea and historical definitions of Mediterranean populations. These definitions traditionally include regions with shared cultural and agricultural practices, particularly olive cultivation across the Mediterranean basin (26, 42). The higher proportion of younger individuals (18–35 years) and females in the NMC cohort contrasts with trends typically reported in Mediterranean populations, where younger demographics often dominate due to higher birth rates and delayed transitions to adulthood (43). However, our findings align with demographic studies highlighting a younger population in non-Mediterranean regions, as well as with global trends where females in these regions are more likely to participate in higher education and the workforce, particularly in Western and Central Europe (44, 45). The present findings suggest that the NMC’s younger demographic may be influenced by selective migration, where younger individuals relocate for education or employment opportunities—a phenomenon well-documented in high-income European nations like Germany and Luxembourg (46). This hypothesis is supported by the high proportion of students and employees in the NMC region studied, compared to the MC. A pattern which may reflect national policies prioritizing access to tertiary education, aimed at improving workforce competitiveness and fostering economic development (43). The greater proportion of single individuals in the NMC further supports this notion, as delayed marriage is often linked to urbanization and economic mobility (47). Conversely, the MC’s higher rate of married/cohabiting individuals aligns with cultural norms that prioritize familial ties, particularly in North African and Southern European societies (48). In contrast, in non-Mediterranean countries, particularly in Western societies, there is a trend toward later marriage and a greater acceptance of non-traditional living arrangements (49).

Regarding the educational and employment disparities, the NMC’s higher bachelor’s degree attainment contrasts with the MC’s greater proportion of master/doctoral degrees. These disparities reveal distinct socioeconomic strategies. The NMC’s higher bachelor’s degree attainment reflects policy emphasis on workforce-ready education, where countries like Germany and Luxembourg have aligned undergraduate programs with industrial demands through dual education systems (50). Conversely, the MC’s greater proportion of advanced degrees signals an adaptive response to economic challenges—graduate education expansion in countries like Spain and Italy functions both as a buffer against youth unemployment (reaching 27.2% in Mediterranean EU nations in 2023) and a mechanism to enhance competitiveness in knowledge-based sectors (43, 51). This educational divergence is mirrored in employment patterns: the MC’s higher employment rate reflects economic necessity driving earlier workforce entry, particularly in service and agricultural sectors where education-occupation mismatches are prevalent (52). Meanwhile, the NMC’s larger student population and higher retirement rates illustrate the demographic and economic privilege of extended education in aging, high-income societies (53, 54). These trends suggest that while NMC regions optimize education for immediate labor market integration, MC nations employ advanced education as both an economic adaptation strategy and social stabilizer amidst unemployment crises.

The observed differences in body weight profiles between Mediterranean (MC) and non-Mediterranean (NMC) populations reflect both persistent dietary advantages and concerning behavioral trends. The MC’s higher prevalence of normal-weight individuals (55.1% vs. 50.6%; *p* < 0.01) and lower overweight rates (39% vs. 42.5%; *p* < 0.05) confirm the enduring protective effects of traditional Mediterranean dietary patterns, characterized by high consumption of olive oil, fish, and plant-based foods (42, 55). However, the narrowing gap suggests nutritional transition, where globalization has increased processed food consumption in MC regions by approximately 27% since 2010, diluting the diet’s protective effects (56). This dietary convergence is particularly evident among younger MC cohorts, who show BMI trends approaching NMC levels. The NMC’s significantly higher smoking abstinence (80.3% vs. 72.1%; *p* < 0.001) demonstrates the success of comprehensive tobacco control policies in countries like Germany, where smoke-free legislation and advertising bans reduced smoking prevalence by 35% between 2005 and 2020 (57, 58). Conversely, the MC’s persistent tobacco use reflects both the cultural entrenchment of shisha in social practices—with 42% of Lebanese and 28% of Tunisian adults reporting regular use (59, 60)—and slower implementation of WHO Framework Convention on Tobacco Control measures in Mediterranean nations. These findings highlight the need for culturally adapted public health strategies that address both emerging nutritional challenges and traditional risk behaviors. The living environment and health status did not show significant regional differences, suggesting that similarities exist between the studied Mediterranean and non-Mediterranean countries in terms of environmental stressors and access to healthcare.

### 4.2 Mediterranean diet and lifestyle adherence and perceived barriers

The regional disparities in MedLife Index scores highlight greater adherence to the Mediterranean diet (MedDiet) and lifestyle among participants from Mediterranean countries (MC) compared to those from non-Mediterranean countries (NMC), reflecting enduring cultural dietary patterns. These findings align with previous research, which has shown that Mediterranean populations are more likely to adhere to the principles of the MedDiet (26, 42). However, these disparities also suggest cultural and economic influences in NMC regions, where access to fresh, healthy Mediterranean foods may be limited or more expensive, leading to a greater reliance on processed foods (61, 62). It is important to note that the modest Cohen’s d value (0.17) for differences in the MedLife Index, despite statistical significance, indicates that while traditional dietary patterns persist in MC regions, their distinctiveness is eroding, likely due to globalization-driven homogenization of food systems (63). While MC populations continue to show higher adherence to core MedDiet elements like fish and legumes, the rise of processed foods and shifting agricultural economies have reduced the historical gaps. This convergence is particularly evident among younger cohorts in MC regions, whose snacking behaviors (with only 65% limiting nibbling) now reflect globalized eating patterns (64).

The regional variations in specific food groups shown in the present findings further support these suggestions. Indeed, MC participants showed superior adherence to traditional MedDiet pillars, including (i) plant-based proteins with higher legume consumption, aligning with the MedDiet’s emphasis on pulses as primary protein sources (42); (ii) lean animal proteins, with greater adherence to fish/seafood and white meat recommendations, consistent with coastal dietary traditions (65); and (iii) sugar moderation, with lower sweets intake, reflecting cultural preferences for fresh fruits as desserts (66). In contrast, NMC participants demonstrated stronger adherence to modern MedDiet adaptations, including (i) meat moderation, with better compliance with limiting red and processed meat, likely influenced by Northern European public health campaigns (67); (ii) whole grains and herbs, with higher cereal and herb/spice use, which align with the MedDiet’s plant-centric ethos and locally available ingredients (68). This dichotomy suggests that while MC regions preserve traditional MedDiet practices rooted in local food systems, they face challenges in adopting newer evidence-based recommendations, such as the reduction of red meat consumption. Conversely, NMC regions appear to adopt MedDiet principles selectively, emphasizing aspects that are compatible with their food environments (e.g., whole grains over seafood), demonstrating the diet’s adaptability beyond its geographic origins (69).

Regarding Mediterranean dietary habits, MC participants scored significantly higher in several categories, including water and wine consumption, and limiting salt intake in meals. These findings reflect the lifestyle choices typically associated with the Mediterranean region, where hydration, moderate alcohol consumption, and low sodium intake are common components of the traditional diet (70). On the other hand, NMC participants reported a stronger preference for whole grains, which aligns with global trends of increasing whole grain consumption in response to growing health awareness (71). However, the higher adherence to whole grains in NMC compared to MC could also be influenced by the broader availability and cultural acceptance of whole grains in non-Mediterranean regions.

The absence of significant regional differences in overall Block 3 scores suggests growing global lifestyle homogenization, yet nuanced behavioral patterns persist. MC participants maintained stronger engagement in culturally rooted social practices, demonstrating higher rates of both socializing with friends and collective sports participation. These findings align with traditional Mediterranean lifestyle models emphasizing communal physical activity and face-to-face interaction as integral to wellbeing (65, 72). The preservation of these practices likely reflects deeper cultural values prioritizing social cohesion, even amidst dietary transitions (64). Conversely, NMC participants exhibited distinct compensatory behaviors: more frequent napping and stricter television time management. While these patterns may initially appear contradictory, they potentially represent adaptive responses to Northern Europe’s productivity-oriented cultures—where napping serves as a stress-regulation strategy in high-demand work environments (73), and conscious screen limitation counters sedentary office lifestyles (74). The MC’s comparatively lower TV restriction may paradoxically reflect stronger outdoor leisure traditions reducing screen dependence (75). These findings collectively suggest that while globalization has diminished overt lifestyle disparities (evidenced by comparable Block 3 scores), region-specific coping mechanisms and cultural priorities continue to shape health behaviors in subtle but meaningful ways. This has important implications for public health strategies, suggesting that MedDiet interventions should leverage existing social networks to reinforce dietary changes through community-based programs in MC contexts and compensatory behaviors like napping as potential gateways to broader lifestyle modification in NMC contexts.

The analysis of perceived barriers reveals distinct yet overlapping obstacles to MedDiet adoption across regions. While no significant difference emerged in overall barrier perception, MC participants faced more pronounced structural challenges. Economic constraints (i.e., price unaffordability) were particularly salient, reflecting inflationary pressures on traditional foods like olive oil and fish most probably in Southern Europe (76). Accessibility barriers were also more prevalent in MC, likely due to urban food deserts limiting fresh produce availability in rapidly modernizing cities (77). Cultural factors further complicated adherence, as 91.6% of MC participants reported belief-related barriers, mirroring generational shifts toward convenience foods despite strong dietary traditions (64). These findings align with prior research showing that even in Mediterranean countries, socioeconomic factors increasingly undermine dietary quality (78). In contrast, NMC participants encountered primarily knowledge-and behavior-related hurdles, suggesting limited familiarity with MedDiet principles in regions without cultural exposure to this eating pattern (71). Practical implementation challenges were equally significant, with time constraints and motivation deficits reflecting the demands of Northern Europe’s fast-paced work culture (74). These behavioral barriers align with the theory of planned behavior, which identifies intention and perceived control as critical factors in dietary adoption (79). Together with knowledge gaps, these obstacles create a dual challenge for NMC populations: comprehending MedDiet principles while adapting them to incompatible lifestyles. Despite these regional differences, the high prevalence of accessibility and belief barriers in both groups (exceeding 84% overall) signals universal challenges in contemporary food environments. The persistence of such obstacles, even in traditional MedDiet heartlands, highlights the need for targeted interventions: price stabilization and retail access improvements in MC regions, paired with education and behavior-change strategies in NMC contexts. Future research should explore how these barriers interact with demographic factors like income and urbanization to better inform policy solutions.

### 4.3 Physical and social activities, sleep patterns, and technology use behavior

Although MC participants exhibited greater engagement in lifestyle-integrated physical activities—such as walking, household chores, and active socializing, as assessed by Block 3 of the MedLife Index—this did not translate into higher overall physical activity levels. Data from the more comprehensive IPAQ questionnaire, which measures total physical activity, including structured exercise, occupational movement, and recreational activities, revealed the absence of significant differences in overall physical activity levels between regions, with 60–62% of participants in both areas engaging in low activity, underscores a global trend more in gavour of sedentarization that transcends cultural boundaries (80). However, the higher proportion of moderate activity in MC suggests residual cultural practices of lifestyle-embedded movement—such as walking for transportation or social purposes—that persist despite broader activity declines (65). This aligns with the Mediterranean lifestyle model that traditionally integrates physical activity into daily routines rather than structured exercise (42), though its protective effects may be weakening in contemporary urban environments.

Regarding the social participation patterns the present findings report a cultural paradox. While MC maintained higher proportions of “sometimes socially active” individuals, NMC showed greater representation in the “always socially active” category and higher SSPQ scores (Cohen’s d = 0.1). This likely reflects divergent social models—MC’s informal, frequent interactions versus NMC’s more organized but less frequent engagements (81). The Mediterranean pattern of sustained but variable social connectivity may derive from cultural norms prioritizing familial and community bonds (64), whereas Northern European societies may favor scheduled social commitments within individualistic frameworks (82). Similarly, sleep analyses uncovered clinically relevant contrasts. Indeed, despite equivalent sleep efficiency and latency, MC participants reported poorer sleep quality and shorter duration, with 45% sleeping below recommended levels versus 40% in NMC. These findings implicate cultural factors like late-night dining (83) and evening-oriented social rhythms (84) as potential contributors. Paradoxically, NMC showed higher insomnia rates (moderate/severe: 25% vs. 19.3%), suggesting that while Mediterranean lifestyles may disrupt sleep quantity, Northern European work cultures might exacerbate sleep disorders through productivity-related stress (73). This dissociation between subjective sleep quality and clinical insomnia warrants further investigation into cultural perceptions of sleep health (85).

Technology use showed striking homogeneity, with ≈80% of both groups reporting frequent-to-constant engagement. This universal digital saturation reflects technology’s role as a cultural equalizer, potentially eroding traditional lifestyle distinctions while creating new health challenges (86). The lack of regional differences contradicts assumptions about the Mediterranean “digital lag” and underscores technology’s pervasive influence on modern lifestyles.

### 4.4 Life satisfaction, mental health (DASS-21), and the need for psychosocial, physical, and nutritional support

Our findings challenge the traditional idea that mental health issues are significantly different across regions. We found no significant differences in life satisfaction, depression, anxiety, or stress between participants from MC and NMC. In both regions, about 33–35% of people reported moderate symptoms, which reflects global trends in mental health after the COVID-19 pandemic, where mental health issues have become widespread across different economic contexts (87). These findings contradict earlier studies that suggested Mediterranean populations suffer from worse mental health due to economic instability (88). This discrepancy could be due to differences in research methods when applying mental health assessments across cultures (89), or perhaps the increasing globalization is contributing to similar levels of psychological distress worldwide due to common digital and economic challenges (90). The present findings suggest that traditional risk factors for mental health, like economic difficulties (26), may be counterbalanced by cultural factors such as strong family support networks (48). Alternatively, it may indicate that economic measures no longer reflect individual psychological well-being in modern welfare states (91).

Surprisingly, participants from the studied NMC in the present study (suggested to have low economic challenges) reported a significantly greater need for both psychosocial and physical support compared to MC. This difference likely reflects three factors: (i) NMC countries have established systems for seeking help, where robust social welfare programs (like Germany’s Pflegeversicherung) make it normal to request support (92); (ii) in MC regions, people tend to rely on informal family support networks, reducing the need for formal assistance (93); and (iii) NMC countries have older populations, which creates more demand for physical care (43). The lower demand for support in MC regions might also reflect underreporting due to stigma—a common issue in collectivist societies, where acknowledging a need for help can risk family honor (94). These findings call for a reassessment of the “Mediterranean paradox,” which suggests that despite economic hardship, Mediterranean countries often show lower formal support utilization. Instead of viewing this as a sign of resilience, it may reflect cultural resourcefulness—where people rely on family and community networks rather than formal services (95). This finding highlights the need to understand cultural contexts better when evaluating support systems and mental health in different regions.

### 4.5 Public health implications

Our findings reveal both the resilience of traditional health behaviors and the growing pressures of modernization across Mediterranean (MC) and non-Mediterranean (NMC) regions. These insights point to three critical pathways for public health action that balance cultural preservation with contemporary adaptation.

#### 4.5.1 Region-specific dietary preservation and promotion

The study’s most urgent policy imperative lies in safeguarding Mediterranean dietary traditions while making them accessible in modern contexts. For MC regions, this requires dual interventions: economic support for traditional food systems through agricultural subsidies (particularly for olive oil, fish, and legumes under CAP reforms) paired with urban planning regulations to combat food deserts (96). Successful models like Barcelona’s “Eat Mediterranean” program demonstrate how municipal kitchens can deliver affordable MedDiet meals while creating local employment (97). Simultaneously, digital platforms could reinvigorate cultural pride in traditional diets among younger generations—Spain’s “Mediterranean Diet Virtual Museum” shows how gamification can make heritage appealing to digital-native youth (98).

In NMC regions, adoption strategies must address the knowledge-motivation gap through behavioral “nudge” techniques. German workplace trials where cafeterias positioned Mediterranean options at eye-level increased selection by 38% without restricting choice (99). Supermarket partnerships could extend this approach through ready-to-cook MedDiet meal kits, addressing time barriers identified in our study. Primary care systems in Northern Europe should integrate dietitian referrals—following the UK’s “Social Prescribing” model that links clinical care with community cooking classes (100).

#### 4.5.2 Mental health system innovation

The paradox of higher formal support requests in NMC despite comparable mental health metrics suggests systemic inefficiencies in addressing mild-to-moderate cases. Stepped-care models piloted in the Netherlands—where digital CBT precedes specialist referral—reduced treatment delays by 60% while maintaining outcomes (101). These could be adapted to Mediterranean contexts by training community health workers as “culture brokers” who connect formal services with existing familial support networks, as successfully trialed in southern Italy’s “Mental Health Bridges” program (102).

#### 4.5.3 Lifestyle integration and digital integration

The universal physical activity decline and technology saturation demand reimagining healthy living in digital societies. Urban design must prioritize “movement-friendly” environments: Barcelona’s “superblocks” initiative increased neighborhood walking by 22% through traffic calming (103), while Copenhagen’s bicycle infrastructure serves as a model for active commuting. For screen-dominated lifestyles, app-based interventions like Greece’s “MedMove” demonstrate how smartphone reminders can convert sedentary time into micro-activity breaks without requiring gym access (104).

By anchoring interventions in cultural strengths while addressing modernization’s challenges, policymakers can develop sustainable approaches to health promotion that respect regional identities while meeting global health targets. The Mediterranean diet’s evolutionary history—adapting over millennia while retaining core principles—offers a powerful metaphor for this balanced approach to 21st century public health.

### 4.6 Limitations

This study possesses multiple strengths, including its multinational design and diverse population (N = 4,010), which enhance the generalizability of findings across cultural contexts. The use of validated assessment tools provides a comprehensive evaluation of demographic, behavioral, and psychosocial factors influencing MedDiet adherence. However, several limitations warrant discussion. First, the online recruitment method, while enabling efficient multinational data collection, may have favored participation from more health-conscious, urban, and technologically adept populations, particularly underrepresenting older adults and rural residents. Furthermore, the sample size disparity between Mediterranean (MC: *n* = 2,594, 64.7%) and non-Mediterranean (NMC: *n* = 1,416, 35.3%) groups represent a significant limitation. While we employed robust statistical methods such as the proportional analyses (focusing on relative distributions (%) rather than absolute counts) and Z-tests for two proportions that explicitly account for unequal group sizes through pooled variance estimation, the imbalance may still reduce precision for detecting small effect size in NMC subgroups. Future studies should prioritize balanced recruitment across regions while maintaining proportional analytical approaches to optimize comparability. Second, cross-sectional design inherently limits causal interpretation of the observed associations between MedDiet adherence and health outcomes. While our findings identify important regional patterns, they cannot establish temporal relationships or directionality. Longitudinal or intervention studies would be valuable to confirm whether improved MedDiet adherence leads to better health outcomes across different cultural contexts. Third, reliance on self-reported data introduces potential measurement biases, including recall bias (particularly for dietary assessments) and social desirability bias. Although we used validated instruments (MedLife Index, IPAQ-SF), the subjective nature of these measures may have affected accuracy. Incorporating objective measures such as biomarkers of dietary intake or device-based physical activity monitoring in future research would strengthen the evidence. Fourth, while we examined numerous covariates, unmeasured confounders may have influenced our results. For instance, variations in local food policies, agricultural practices, or genetic predispositions across regions could affect both dietary patterns and health outcomes but were not accounted for in our analyses. Additionally, the study did not assess potential mediators (e.g., food insecurity, cultural identity) that might explain regional differences in MedDiet adherence. Fifth, our country-level grouping (MC vs. NMC) may have masked important within-region heterogeneity. For example, significant cultural and socioeconomic differences exist between Southern European (e.g., Spain, Italy) and North African (e.g., Tunisia, Morocco) Mediterranean countries that were not explored in depth. Similarly, non-Mediterranean countries like Germany and Luxembourg may differ substantially in food environments and health policies. Future research with larger samples could examine these subgroups separately to identify more nuanced patterns. Finally, the study’s recruitment methods, primarily through online platforms and social networks, may have introduced selection bias toward more health-conscious or technologically adept populations. This could limit the generalizability of findings to broader populations, particularly in regions with lower internet penetration or among older adults less likely to participate in online surveys.

## 5 Conclusion

This multinational study reveals both persistent cultural patterns and converging trends in MedDiet and lifestyle adherence across MC and NMC regions. While significant differences persist in dietary practices—with MC populations maintaining stronger traditional food consumption but facing erosion from processed foods, and NMC regions selectively adopting MedDiet components—our findings demonstrate surprising similarities in mental health outcomes and physical activity levels. These results underscore how globalization is reshaping health behaviors while cultural resilience preserves key dietary distinctions. These findings suggest that public health strategies must be tailored to each region’s cultural, economic, and environmental contexts, promoting MedDiet adherence while addressing barriers such as economic constraints, lifestyle incompatibility, and psychological support needs. Future research should explore the complex relationships between diet, physical activity, mental health, and social support, while also examining the influence of cultural and socio-economic factors on dietary behaviors. This will help develop tailored interventions that address region-specific challenges, considering the role of digital tools in shaping health outcomes. By respecting regional identities while addressing shared modern challenges—from economic pressures to digital lifestyle shifts—public health strategies can preserve the MedDiet’s proven benefits while ensuring its relevance in modern society.

## Data Availability

The datasets generated and analyzed during the current study are not publicly available at this time as further analyses are ongoing, and additional publications based on these data are in preparation. Data may be made available upon reasonable request to the corresponding author once all planned analyses and publications are completed.

## Footnotes

^1^ www.soscisurvey.de/en/privacy
